# Comorbidity between HTLV-1-associated adult T-cell lymphoma/leukemia and verrucous carcinoma: a case report

**Published:** 2017-03-30

**Authors:** Miller Valencia, Luis Moreno

**Affiliations:** Escuela de Medicina Facultad de Salud, Universidad del Valle Cali, Colombia; Hospital Universitario del Valle "Evaristo Garcia" Cali, Colombia

**Keywords:** Leukemia-Lymphoma, adult T-Cell, human T-lymphotropic virus 1, lymphoma, T-cell, carcinoma, verrucous

## Abstract

**Background:**

Adult T-cell Leukemia/Lymphoma (ATLL) is classified as a peripheral CD4+ T-cell neoplasm caused by the human T-cell lymphotropic virus type 1 (HTLV-1). Typical symptoms are associated with leukemic infiltration; however, atypical and exaggerated manifestations of verrucous carcinoma have also been described.

**Case report:**

We present here the case of a patient with multiple skin lesions, ischemic necrosis in the hallux and lymphadenopathies. Biopsies were taken, which showed verrucous epidermal carcinoma and cutaneous lymphoma. Splenomegaly and adenopathy in mesentery, retro peritoneum and lymph node chains in the limbs were observed. Bone marrow examination showed findings compatible with T-cell leukemia/lymphoma; and it was ELISA positive for HTLV-1/2.

**Treatment and outcome:**

The patient had a good initial response to a CHOP scheme (cyclophosphamide, doxorubicin, vincristine and prednisone) with filgrastim. However, the patient had a relapse and died before the second cycle.

**Clinical relevance:**

Comorbidity could lead to the associated risk factors model. According to this model, secondary immunodeficiency caused by HTLV-1 may induce the development of verrucous carcinomas; alternatively, the disease could be due to a correlation between HTLV-1 and the human papillomavirus (HPV).

## Resumen

**Antecedentes:**

La leucemia/linfoma de células T del adulto se clasifica como una neoplasia de células T CD4+ periféricas desencadenada por el virus linfotrópico de células T humanas tipo 1 (HTLV-1). Los síntomas típicos se asocian a la infiltración leucémica; sin embargo, también se ha descrito la manifestación atípica y exagerada de carcinomas verrucosos.

**Caso clínico:**

Se presenta el caso de una paciente con lesiones múltiples en piel, necrosis isquemica del hallux y linfadenopatías. se realizaron biopsias que reportaron carcinoma epidermoide verrugoso y linfoma cutáneo. Se detectó esplenomegalia y adenopatías en mesenterio, región retroperitoneal, y cadenas ganglionares de las extremidades. la medula ósea presentó hallazgos compatibles con leucemia/linfoma de células T y ELISA positivo para HTLV-1/2.

**Tratamiento y resultado:**

La paciente presentó buena respuesta inicial al esquema CHOP (ciclofosfamida, doxorrubicina, vincristina, prednisona) con filgrastim. Sin embargo, presentó recaída de la enfermedad antes del segundo ciclo y falleció

**Relevancia clinica:**

La comorbilidad podría conducir al modelo de factores de riesgo asociados. De acuerdo con este modelo, la inmunodeficiencia secundaria causada por HTLV-1 puede inducir el desarrollo de carcinomas verrucosos; Alternativamente, la enfermedad podría deberse a una correlación entre el HTLV-1 y el virus del papiloma humano (VPH).

## Introduction

Adult T-cell Leukemia/Lymphoma (ATLL) is a peripheral CD4+ T-cell neoplasm caused by human T-cell lymphotropic virus type 1 (HTLV-1) virus ^1^. Adult T-cell Leukemia/Lymphoma is a potentially aggressive type of non-Hodgkin lymphoma ^2^. Adult T-cell Leukemia/Lymphoma occurs in less than 5% of people who are infected with HTLV-1 ^3^. Persons of African heritage have a higher risk than others, and the disease is slightly more common in men than in women. The median age at diagnosis is in the sixth decade. However, median age at diagnosis can vary with geographic location ^4^ ^,^ ^5^.

HTLV-1 infection is endemic in several islands in southern Japan, the Caribbean basin, Western Africa, northeastern Iran and the southeastern United States ^2^ ^,^ ^6^ ^,^ ^7^. With regard to Latin America, the prevalence of HTLV-1 is close to 2% in Argentina, Brazil, Peru and Colombia ^8^. In Colombia, the prevalence rates of HTLV-1 and ATLL were reported to be higher on southern Colombia's Pacific coast, where seroprevalence could reach 5.1% among black people from Tumaco ^9^. There is also a high prevalence of HTLV-1 infection in the Caribbean Region of Colombia, mainly in the department of Córdoba, although with low incidence of ATLL ^9^.

The clinical presentation of HTLV-1-associated ATLL is still being characterized. Secondary findings to leukemic infiltration are described, and an uncommon and excessive expression of verrucous carcinomas can be observed.

## A clinical case report 

This case report describes a 52-year-old woman from the coast of Nariño (Colombia). The patient presented with a 10-year history of progressive development of intensely pruritic papules, plaques and nodules. Symptoms started on the neck and thereafter slowly became widespread. No relevant associated family history was reported.

**Figure 1:**
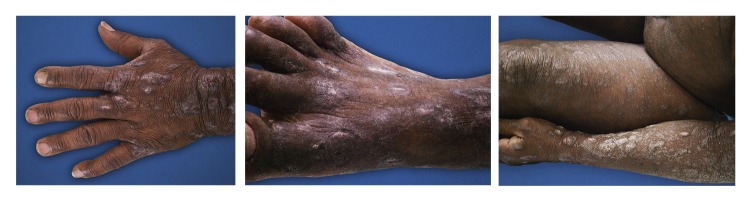
Pruritic papules, plaques and infiltrated nodules in the thorax, abdomen and extremities.

**Figure 2:**
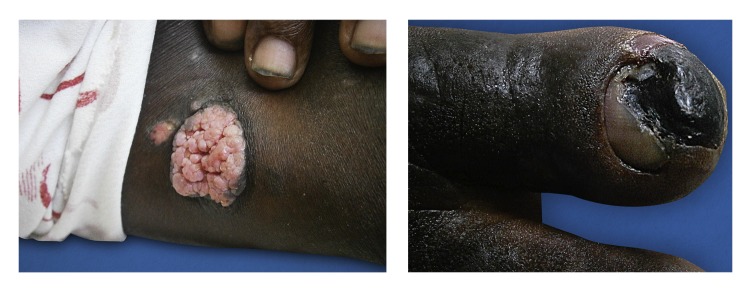
An exophytic papillomatous tumor in the left femoral region (A) and dry necrosis distal from the right hallux (B).

During the physical exam, generalized xerosis, multiple and confluent excoriations, papules, plaques and nodules were observed. Lesions were found on the nape of the neck, anterior thorax, abdomen and extremities, including the palms and soles (Fig. 1). The face and mucous membranes were not involved. Additionally, the patient presented with a bigger lesion in the left femoral region. This lesion was an exophytic, papillomatous, verrucous tumor (Fig. 2A).

The patient reported pain on right hallux that started a week prior to the professional examination. The exam revealed a dry necrotic lesion (^Fig.^B). 

A complete blood count reported 52,800 leukocytes, of which 67.39% were lymphocytes, 11.92% were neutrophils and 15.19% were eosinophils. Determined values for hemoglobin, hematocrit and platelets were 12.16%, 40.43% and 281, respectively. Biochemical assessment showed an increase in lactate dehydrogenase activity (LDH= 833 mg/dL), although renal and hepatic function test results were normal. Anti-HTLV-1/2 titer, signal to cut-off (S/CO) ratio, was measured by chemiluminescent microparticle immunoassay (CMIA). High positive (156.30 S/CO) ratios were obtained for HTLV-1/2. Human immunodeficiency virus (HIV) and Venereal Disease Research Laboratory (VDRL) tests were negative. A direct potassium hydroxide (KOH) mount of a skin scraping showed negative results for mycosis and scabies.

**Figure 3:**
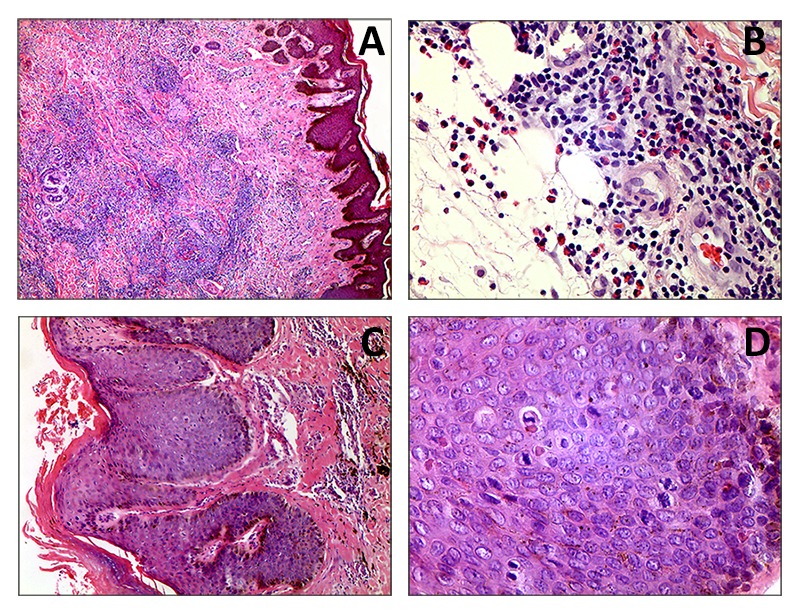
A (4x) y B (40x). A malignant neoplasm lesion of lymphoid origin formed by groups of cells with monotonous, small-lobed nuclei infiltrating into the dermis, accompanied by a moderate number of eosinophils. In some areas, hyperplastic epithelium with Pautrier microabscesses can be observed. C (10X) y D (40X). Skin compromised by a malignant neoplasm lesion of epithelial origin, formed by papillomatous epidermal acanthosis, with orthokeratotic hyperkeratosis and infiltrating tracts of epidermoid differentiated cells. These infiltrated cells possess ovoid nuclei, some with dispersed chromatin and some presenting anisonucleosis, perinuclear cytoplasmic vacuolization and possible Bowenoid transformation, which may suggest a viral etiology. The mitotic activity of the lesion is frequent. Adjacent stroma presented with moderate chronic inflammatory infiltrate.

Biopsies of infiltrated plaques were taken from both upper extremities and from the right leg. These biopsies demonstrated cutaneous lymphoma (Fig. 3 A and B).

Biopsies from exophytic tumors from the left femoral region and the right knee both demonstrated verrucous carcinomas (Figure 3 C y D).

Doppler ultrasound of the lower extremities was performed due to the involvement of the right hallux. Ultrasound revealed the absence of flow in the anterior and posterior tibial arteries and the dorsalis pedis and plantar arteries.

X-ray computed tomography (CT) was performed for the neck and thoracoabdominal regions. The scanner revealed many adenopathies involving several lymph node chains in the neck, armpits, inguinal region, retroperitoneum and mesentery. Splenomegaly was also observed.

A bone marrow biopsy revealed normal trabecular bone, 65% of total marrow cellularity in separate marrow spaces, myeloid hyperplasia and severe eosinophilia. Flow cytometry of the bone marrow reported 48.4% pathological cells with CD4+, CD25+, CD45+, weak sCD3, CD5++ and 10% of CD7+ cells.

These results led to the diagnosis of HTLV-1 infection-associated ATLL during the acute stage associated with a verrucous subtype of squamous cell carcinoma. Treatment with polychemotherapy was initiated after concluding that the patient had an unresectable cancer. The patient received a combination of CHOP (1200 mg cyclophosphamide, 80 mg doxorubicin, 2 mg vincristine, 100 mg prednisone) and polyethylene glycol (PEG) filgrastim (6 mg PEG filgrastim). The initial response to polychemotherapy was favorable; however, the patient had a subsequent relapse and died before the second cycle.

## Discussion

Adult T-cell Leukemia/Lymphoma is associated with all cases of HTLV-1 infection. HTLV-1 plays a central role in ATLL pathogenesis. The HTLV-1 genome is integrated into the genome in T cells prior to viral replication ^10^. The viral nuclear protein Tax induces oncogenesis in HTLV-1-infected T cells by decreasing BCL11B tumor suppressor protein expression ^11^.

Squamous cell carcinoma presents a broad variety of clinical manifestations, including papules, plaques, hyperkeratotic nodules and ulcerative lesions. These lesions can be developed on any skin surface. Verrucous carcinoma is a subtype of squamous cell carcinoma presenting with exophytic, cauliflower-like lesions similar to large warts ^12^.

The patient age fell in the more frequent range for ATLL. The patient was likely infected at a young age through either breastfeeding or sexual intercourse. The patient's chronic ailment, which began as an infective dermatitis, is remarkable.

During adulthood, the patient had presented with the reported lesions for more than a decade. Without proper handling of the lesions, they transformed into malignant neoplasms that consisted of multiple verrucous carcinomas. It is possible that the exact reason for this transformation can be discovered.

The concept of comorbidity leads to a possible direct causation model. According to this model, a chronic skin infiltration during ATLL will be responsible for verrucous carcinomas ^13^.

However, the concept of comorbidity could lead to the associated risk factors model. According to this model, secondary immunodeficiency caused by HTLV-1 may induce the development of verrucous carcinomas; alternatively, the disease could be due to a correlation between HTLV-1 and human papillomavirus (HPV)^13^. It is known that HTLV-1 may worsen the clinical course of infections with other pathogens, including other viruses, such as HPV ^14^ ^,^ ^15^.

Persistent infection with HPV has been identified as a cofactor for the development of squamous cell carcinoma. The clinical and histopathological findings of verrucous carcinomas can be correlated with a permanent state of HPV infection, which can persist due to HTLV-1 infection. The persistence of verrucous carcinomas is facilitated by abnormalities in CD4+ T cells ^16^. It has been reported that HTLV-1 is associated with the development of some secondary malignancies to massive leukemic cell infiltration ^17^ ^,^ ^18^. However, it is not clear if HTLV-1 itself can develop generalized verrucous carcinomas, which are usually associated with HPV.

Zidovudine and interferon-α are promising treatments for this type of disease ^2^. Allogeneic hematopoietic stem cell transplantation has also been included as a treatment modality. The anti-CCR4 monoclonal antibody mogamulizumab has cytotoxic effects on ATLL cells, although the response rate may be improved by combining mogamulizumab with chemotherapy during the acute stage ^19^.

A Tax peptide-pulsed dendritic cell vaccine is a promising immunotherapy for ATLL remission at 2 months and for maintaining the remission for 2 years after vaccination ^20^.

## Conclusion

This case report described a patient diagnosed with a HTLV-1 infection-associated ATLL in addition to verrucous carcinoma. 

Verrucous carcinoma is a subtype of squamous cell carcinoma with distinctive clinical and histopathological features. One of its risk factors is immunosuppression secondary to lymphomas; in addition, it can develop as ulcers on chronic inflammatory dermatoses. These two risk factors are present in the case (described here), so we consider that the immunosuppression caused by lymphoma and the chronic ulcerated lesions caused by HTLV infection facilitated the appearance of verrucous carcinoma. Preventing the virus from passing from mother to child is a key issue for the eradication of this disease.

## References

[1] Swerdlow SH, Campo E, Harris NL, Jaffe ES, Pileri SA, Stein H (2008). WHO classification of tumours of haematopoietic and lymphoid tissues. World Health Organization classification of tumours.

[2] Ma W-L, Li C-C, Yu S-C, Tien H-F (2014). Adult T-Cell Lymphoma/Leukemia Presenting as Isolated Central Nervous System T-Cell Lymphoma. Case Reports in Hematology.

[3] Shimoyama M (1991). Diagnostic criteria and classification of clinical subtypes of adult T-cell leukaemia-lymphoma A report from the Lymphoma Study Group (1984-87). Br J Haematol.

[4] Vose J, Armitage J, Weisenburger D (2008). International peripheral T-cell and natural killer/T-cell lymphoma study pathology findings and clinical outcomes. J Clin Oncol.

[5] Matutes E (2007). Adult T-cell leukaemia/lymphoma. J Clin Pathol.

[6] Bitar N, Hajj HE, Houmani Z, Sabbah A, Otrock ZK, Mahfouz R, Zaatari G, Bazarbachi A (2009). Adult T-cell leukemia/lymphoma in the Middle East first report of two cases from Lebanon. Transfusion.

[7] Chihara D, Ito H, Matsuda T, Shibata A, Katsumi A, Nakamura S (2014). Differences in incidence and trends of haematological malignancies in Japan and the United States. Br J Haematol.

[8] Salcedo-Cifuentes M, Restrepo O, Garcia-Vallejo F (2011). Epidemiología y bioinformática en el estudio de la Leucemia Linfoma de Células T del Adulto asociada a la infección con VLHT-1. Rev Salud Publica (Bogota).

[9] Carrascal E, Cortés A, Akiba S, Tamayo O, Quiñónez F, Flórez L (2004). Epidemiología y patología de la leucemia/linfoma de células T del adulto en Cali y el suroccidente colombiano. Colomb Med.

[10] Baydoun H, Duc-Dodon M, Lebrun S, Gazzolo L, Bex F (2007). Regulation of the human T-cell leukemia virus gene expression depends on the localization of regulatory proteins Tax, Rex and p30II in specific nuclear subdomains. Gene.

[11] Takachi T, Takahashi M, Takahashi-Yoshita M, Higuchi M, Obata M, Mishima Y (2015). Human T-cell leukemia virus type 1 Tax oncoprotein represses the expression of the BCL11B tumor suppressor in T-cells. Cancer Sci.

[12] Yanofsky VR, Mercer SE, Phelps RG (2011). Histopathological variants of cutaneous squamous cell carcinoma a review. J Skin Cancer.

[13] Valderas JM, Starfield B, Sibbald B, Salisbury C, Roland M (2009). Defining comorbidity implications for understanding health and health services. Ann Fam Med.

[14] Tokunaga M, Uto H, Oda K, Mawatari S, Kumagai K, Haraguchi K (2014). Influence of human T-lymphotropic virus type 1 coinfection on the development of hepatocellular carcinoma in patients with hepatitis C virus infection. J Gastroenterol.

[15] Lopo SS, Oliveira PM, Santana IU, Pena GB, Torrales MB, Mascarenhas RE (2012). Evidence of a higher prevalence of HPV infection in HTLV-1-infected women a cross-sectional study. Rev Soc Bras Med Trop.

[16] Andersen AS, Koldjaer Solling AS, Ovesen T, Rusan M (2014). The interplay between HPV and host immunity in head and neck squamous cell carcinoma. Int J Cancer.

[17] Khadilkar UN, Mathai AM, Chakrapani M, Prasad K (2010). Rare association of papillary carcinoma of thyroid with adult T-cell lymphoma/leukemia. Indian J Pathol Microbiol.

[18] Miyahara H, Itou H, Sekine A, Taniyama D, Katsui T, Tanaka W (2009). A case of adult T-cell leukemia/lymphoma with primary lung cancer. Nihon Kokyuki Gakkai Zasshi.

[19] Utsunomiya A, Choi I, Chihara D, Seto M (2015). Recent advances in the treatment of adult t-cell leukemia- lymphomas. Cancer Sci.

[20] Suehiro Y, Hasegawa A, Iino T, Sasada A, Watanabe N, Matsuoka M (2015). Clinical outcomes of a novel therapeutic vaccine with Tax peptide-pulsed dendritic cells for adult T cell leukaemia/lymphoma in a pilot study. Br J Haematol.

