# Supplementary material for: Comorbidity between HTLV-1-associated adult T-cell lymphoma/leukemia and verrucous carcinoma: a case report
Source: Colomb Med (Cali). 2017 Mar 30;48(1):35–8. (PMC5438227)
Supplement: Translation

# Translation: Comorbilidad entre carcinoma verrugoso y linfoma/leucemia de células T del adulto asociado con HTLV-1: un reporte de caso

## Introducción

La leucemia/linfoma de células T del adulto se clasifica como una neoplasia de células T CD4+ periféricas desencadenada por el virus linfotrópico de células T humanas tipo 1 (HTLV-1) ^1^. Es una variante del linfoma no Hodgkin de células T y puede ser altamente agresiva ^2^. Tiene una incidencia menor del 5% en personas infectadas con el HTLV-1 ^3^, es más común en la raza negra que en otras razas y se describe un ligero predominio en los hombres. La edad media del diagnóstico es en la sexta década; sin embargo, puede variar por la ubicación geográfica ^4^^,^^5^.

La infección es endémica en varias islas del sur de Japón, la Cuenca del Caribe, África occidental, el noreste de Irán y el sureste de los Estados Unidos ^2^^,^^6^^,^^7^. En Latinoamérica, el HTLV-1 se reporta con prevalencias cercanas al 2% en Argentina, Brasil, Perú y Colombia ^8^.

En Colombia se ha registrado el virus y la enfermedad con mayor prevalencia en el sur de la costa pacífica colombiana, alcanzado cifras de serología positiva en un 5,1% de la población negra del municipio de Tumaco ^9^. También, hay una prevalencia de población infectada por el HTLV-1 en la región caribe, especialmente en el departamento de Córdoba, aunque con baja incidencia de la enfermedad ^9^.

La presentación clínica de los pacientes con leucemia/linfoma de células T del adulto asociado a HTLV-1 aún continúa caracterizándose. Exponemos como, además de los hallazgos más conocidos secundarios a la infiltración leucémica, se puede exhibir una manifestación atípica y exagerada de carcinomas verrucosos.

## Caso clínico

 Este reporte de caso describe a una mujer de 52 años de la Costa (Pacífica) de Nariño (Colombia). La paciente presentó una historia de 10 años de desarrollo progresivo de pápulas, placas y nódulos intensamente pruriginosos. Los síntomas comenzaron en el cuello y luego se extendieron lentamente. No se informó de ningún antecedente familiar asociado relevante.

 Durante el examen físico se observaron escoriaciones múltiples y confluentes, xerosis generalizada, pápulas, placas y nódulos. Se encontraron lesiones en la nuca, el tórax anterior, el abdomen y las extremidades, incluyendo las palmas de las manos y las plantas de los pies (Fig. 1). La cara y las membranas mucosas no estuvieron involucradas. Además la paciente presentó una lesión más grande en la región femoral izquierda. Esta lesión era un tumor exofítico, papilomatoso, verrugoso (Fig. 2A).

**Figura 1:** Pápulas pruriginosas, placas y nódulos infiltrados en el tórax, el abdomen y extremidades.

**Figura 2:** Un tumor papilomatoso exofítico en la región femoral izquierda y necrosis distal seca del hallux derecho.

 La paciente informó dolor en el hallux derecho, el cual comenzó una semana antes del examen médico. El examen reveló una lesión necrótica seca (Fig. 2B). 

 Un recuento sanguíneo completo informó 52,800 leucocitos, de los cuales 67,39% eran linfocitos, 11.92% eran neutrófilos y 15.19% eran eosinófilos. Los valores determinados para hemoglobina, hematocrito y plaquetas fueron 12.16%, 40.43% y 281, respectivamente. La evaluación bioquímica mostró un aumento en la actividad de la lactato deshidrogenasa (LDH= 833 mg/dL), aunque los resultados de las pruebas de función renal y hepática fueron normales. Para los títulos de anticuerpos anti-HTLV - 1/2, se midió la relación señal a punto de corte (S/CO) mediante el método de inmunoensayo de quimioluminiscencia de micro-partículas (CMIA). Se obtuvieron relaciones positivas altas (156.30 S/CO) para HTLV-1/2. Las pruebas del virus de inmunodeficiencia humana (VIH) y las pruebas para enfermedades de transmisión sexual (ETS) fueron negativas. Un examen directo de un raspado de piel con hidróxido de potasio (KOH) mostró resultados negativos para micosis y escabiosis. 

 Se tomaron biopsias de placas infiltradas de ambas extremidades superiores y de la pierna derecha. Estas biopsias demostraron linfoma cutáneo (Fig 3, A y B).

**Figura 3:** A (4X), B (40X): Una lesión neoplásica maligna de origen linfoide, formada por grupos de células con núcleos monótonos de lóbulo pequeño, las cuales se infiltran en la dermis, acompañadas por un número moderado de eosinófilos. En algunas áreas se puede observar epitelio hiperplásico con microabscesos de Pautrier. C (10X), D (40X): Piel comprometida por una lesión neoplásica maligna de origen epitelial, formada por acantosis epidérmica papilomatosa, con hiperqueratosis ortoqueratótica y tractos infiltrantes de células epidermoides diferenciadas. Estas células infiltradas poseen núcleos ovoides, algunos con cromatina dispersa; y algunos presentan anisonucleosis, vacuolización citoplasmática perinuclear y posible transformación Bowenoide, lo cual puede sugerir una etiología viral. La actividad mitótica de la lesión es frecuente. El estroma adyacente presenta infiltrado inflamatorio crónico moderado.

 Las biopsias de los tumores exofíticos de la región femoral izquierda y la rodilla derecha demostraron ambas carcinomas verrugosos (Fig. 3, C y D).

 La ecografía Doppler de las extremidades inferiores se realizó debido al compromiso del hallux derecho. El ultrasonido reveló ausencia de flujo en las arterias tibial anterior y posterior y en las arterias dorsal y plantar del pie.

 Se realizó una tomografía computarizada (TC) de rayos X para las regiones cervical y toracoabdominal. El escáner reveló muchas adenopatías que comprometían varias cadenas de ganglios linfáticos en el cuello, axilas, región inguinal, retroperitoneo y mesenterio. También se observó esplenomegalia.

 Una biopsia de médula ósea reveló hueso trabecular normal, el 65% de la celularidad total de la médula en espacios medulares separados, hiperplasia mieloide y eosinofilia severa. La citometría de flujo de la médula ósea informó un 48,4% de células patológicas con CD4 +, CD25 +, CD45 +, sCD3 débil, CD5 ++ y 10% de células CD7 +.

 Estos resultados condujeron al diagnóstico de ATLL asociado a infección por HTLV-1 en fase aguda, asociado con un subtipo verrugoso de carcinoma de células escamosas. Se inició tratamiento con poliquimioterapia después de concluir que la paciente tenía un cáncer no resecable. La paciente recibió una combinación de CHOP (1200 mg de ciclofosfamida, 80 mg de doxorrubicina, 2 mg de vincristina, 100 mg de prednisona) y polietilenglicol (PEG) filgrastim (6 mg de PEG filgrastim). La respuesta inicial a la poliquimioterapia fue favorable; sin embargo, la paciente tuvo una recaída posterior y murió antes del segundo ciclo.

## Discusión

La leucemia/linfoma de células T del adulto se asocia en todos los casos con la infección por HTLV-1, desempeñando este virus un papel cardinal en su patogénesis. El virus se incorpora en el genoma de las células T antes de su expansión clonal ^10^. La proteína nuclear viral Tax promueve la oncogénesis de células T infectadas reduciendo la expresión de la proteína supresora de tumores BCL11B ^11^.

 El carcinoma de células escamosas presenta una amplia variedad de manifestaciones clínicas, incluyendo pápulas, placas, nódulos hiperqueratósicos y lesiones ulcerosas. Estas lesiones se pueden desarrollar en cualquier superficie de la piel. El carcinoma verrugoso es un subtipo de carcinoma de células escamosas que presenta lesiones exofíticas similares a grandes verrugas ^12^.

 La edad de la paciente está en el rango más frecuente para la ATLL. La paciente probablemente se infectó a una edad temprana a través de la lactancia materna o las relaciones sexuales. La enfermedad crónica de la paciente, que comenzó como una dermatitis infecciosa, es notable.

 Durante la edad adulta, la paciente había presentado las lesiones reportadas durante más de una década. Sin un manejo adecuado, las lesiones se transformaron en neoplasmas malignos que consistían en múltiples carcinomas verrugosos. Es posible que la razón exacta de esta transformación pueda ser descubierta.

 El concepto de comorbilidad conduce a un posible modelo de causación directa. De acuerdo con este modelo, una infiltración crónica de la piel durante la ATLL sería responsable de carcinomas verrugosos ^13^ Sin embargo, el concepto de comorbilidad podría conducir al modelo de factores de riesgo asociados. De acuerdo con este modelo, la inmunodeficiencia secundaria causada por HTLV-1 puede inducir el desarrollo de carcinomas verrugosos. Alternativamente, la enfermedad podría deberse a una correlación entre el HTLV-1 y el virus del papiloma humano (VPH) ^13^ Se sabe que el HTLV-1 puede empeorar el curso clínico de infecciones con otros patógenos, incluyendo otros virus, como el VPH ^14^^,^^15^.

 La infección persistente con VPH se ha identificado como un cofactor para el desarrollo de carcinoma de células escamosas. Los hallazgos clínicos e histopatológicos de los carcinomas verrugosos pueden estar correlacionados con un estado permanente de infección por VPH, que puede persistir debido a la infección por HTLV-1. La persistencia de los carcinomas verrugosos es facilitada por anomalías en células T CD4+ ^16^. Se ha informado que el HTLV-1 está asociado con el desarrollo de algunas neoplasias malignas secundarias a la infiltración masiva de células leucémicas ^17^^,^^18^. Sin embargo, no está claro si el propio HTLV-1 puede desarrollar carcinomas verrugosos generalizados, los cuales suelen asociarse con el VPH.

 La zidovudina y el interferón-α son tratamientos prometedores para este tipo de enfermedad ^2^. El trasplante alogénico de células madre hematopoyéticas también se ha incluido como una modalidad de tratamiento. El anticuerpo monoclonal anti-CCR4 mogamulizumab tiene efectos citotóxicos en las células ATLL, aunque la tasa de respuesta puede mejorarse combinando mogamulizumab con quimioterapia durante la fase aguda ^19^. 

La vacuna de células dendríticas pulsadas con péptido Tax es una inmunoterapia prometedora por remisión de la enfermedad a los 2 meses y el mantenimiento hasta los 2 años ^20^.

 Una vacuna de células dendríticas pulsadas con péptido Tax es una inmunoterapia prometedora para la remisión de la ATLL a los 2 meses, y para mantener la remisión durante 2 años después de la vacunación ^17^^,^^18^.

## Conclusión

 Este reporte de caso describió a una paciente diagnosticada con ATLL asociada a infección por HTLV-1, además de carcinoma verrugoso.

El carcinoma verrugoso es un subtipo de carcinoma de células escamosas con rasgos distintivos clínicos e histopatológicos. Uno de sus factores de riesgo es la inmunosupresión secundaria a linfomas; además, puede desarrollarse como úlceras en dermatosis inflamatorias crónicas. Estos dos factores de riesgo están presentes en el caso (descrito aquí), por lo que se considera (por lo que consideramos) que la inmunosupresión causada por el linfoma y las lesiones ulceradas crónicas causadas por la infección HTLV facilitaron la aparición del carcinoma verrugoso. Prevenir el paso del virus de la madre al hijo es un asunto clave para la erradicación de esta enfermedad.
